# Innovations in curriculum design: A multi-disciplinary approach to teaching statistics to undergraduate medical students

**DOI:** 10.1186/1472-6920-8-28

**Published:** 2008-05-01

**Authors:** Jenny V Freeman, Steve Collier, David Staniforth, Kevin J Smith

**Affiliations:** 1 School of Health and Related Research, University of Sheffield, Sheffield, UK; 2 Learning and Teaching Service, University of Sheffield, Sheffield, UK; 3 Independent Educational Consultant, Sheffield, UK

## Abstract

**Background:**

Statistics is relevant to students and practitioners in medicine and health sciences and is increasingly taught as part of the medical curriculum. However, it is common for students to dislike and under-perform in statistics. We sought to address these issues by redesigning the way that statistics is taught.

**Methods:**

The project brought together a statistician, clinician and educational experts to re-conceptualize the syllabus, and focused on developing different methods of delivery. New teaching materials, including videos, animations and contextualized workbooks were designed and produced, placing greater emphasis on applying statistics and interpreting data.

**Results:**

Two cohorts of students were evaluated, one with old style and one with new style teaching. Both were similar with respect to age, gender and previous level of statistics. Students who were taught using the new approach could better define the key concepts of p-value and confidence interval (p < 0.001 for both). They were more likely to regard statistics as integral to medical practice (p = 0.03), and to expect to use it in their medical career (p = 0.003). There was no significant difference in the numbers who thought that statistics was essential to understand the literature (p = 0.28) and those who felt comfortable with the basics of statistics (p = 0.06). More than half the students in both cohorts felt that they were comfortable with the basics of medical statistics.

**Conclusion:**

Using a variety of media, and placing emphasis on interpretation can help make teaching, learning and understanding of statistics more people-centred and relevant, resulting in better outcomes for students.

## Background

As Medicine has moved to become more evidence based, Medical Statistics has become ever more important and relevant both to the practice of medicine and the education of tomorrow's doctors. However, it is common for students across all disciplines to dislike and under-perform in courses involving mathematics, numeracy or statistics [1,2]. Medical students have varied backgrounds and it is increasingly clear that teaching statistics in a traditional didactic way neither engages the students nor meets their needs; comments from previous student evaluations at our institution have included:

'Medical statistics – YAWN!!! - could be taught in a more interesting way'

'I did not really find the module valuable, as a lot of the content seemed unimportant and difficult to understand'

'Medical statistics lectures were poorly presented and not explained well'

'Statistics sessions were not well presented, too rushed, no handouts'

*'it was difficult to know exactly what we needed to know'*.

Many students fail to understand even the basic concepts, which impacts negatively on their use and interpretation of statistics. This is in contrast with the growing awareness of the importance of statistics to current medical practice, dating back to the 1993 General Medical Council report *Tomorrow's Doctors *which recommended that medical education be required to promote 'the critical evaluation of evidence' [3], something which is impossible without at least a basic knowledge of statistics. Nowadays medical students need to be able to interpret statistics so that they can use them to appropriately inform their medical practice, both during training and most importantly post-qualification when they will be treating patients. The education and training that medical students receive is fundamental to the formation of their attitudes and behaviours. Doctors increasingly need to use statistics to help them understand medical issues. However, this has come at a time when statistics teaching is being squeezed out of an already crowded curriculum and in recent years there has been a move away from teaching techniques towards the teaching of concepts, so as to best utilise the time available [4].

In designing, or indeed redesigning any new module, it will be more effective if its designer determines from the start what it is that they really want the students to know and do [5]. In addition to this consideration it is important to be aware that different students learn in different ways. There are many acknowledged models of learning, for example information processing models which focus on the way that students sense, think, solve problems and remember information [6], or personality models which focus on attention, emotion and values [7]. The fact that there are differences in individual learning styles, however they may be categorized, needs to be appreciated when redesigning any module. Thus, within the limits of what may be possible with regards to time and resources, as wide a variety of approaches as are practicable should be employed

### Educational setting

The current annual intake to the medical undergraduate degree programme in our institution is 260 students. It is a clinically led programme and currently students received only 10 hours of instruction in medical statistics, delivered in their first year. Unless they elect to do a research year, they receive no further training in statistics during their undergraduate training. Thus the challenge is to give students enough instruction to ensure that they understand the basics well, without overloading them with unnecessary detail, within the limited time available.

We describe below an evaluation of a project to reconceptualise the teaching of medical statistics to undergraduate medical students as a result of concerns expressed both by lecturers involved in the teaching and students studying the course. The old course used lectures followed by large group seminars (32 students per group). It was a mathematically-orientated course with much theory and formalae. It was an unpopular and little understood part of the students' early medical education. There was little attempt to contextualize the learning and students often failed to comprehend the relevance of the teaching to both their education and their future practice as medical professionals. We recognized this as a key area for improvement and following a successful bid for institutional teaching and learning development funds, we set about revamping the way in which statistics was taught, in order to make the subject more relevant to the students.

## Methods

### Project description

The project was concerned with redesigning the delivery of medical statistics teaching to medical undergraduates, in order to make it more relevant to their future learning and medical practice. The project offered an opportunity to develop new approaches and materials that would contextualize learning and strengthen the application of statistics to medicine, as recommended by Yilmaz [8], who emphasized the importance of real-world examples when teaching statistics to non-specialists. In line with Garfield's [1995] recommendations we initially concentrated on what it was that we felt students needed to know and be able to do following the teaching sessions. Students of statistics can generally be classified into two groups, those who 'consume' statistics and those who 'do' statistics [9,10]. Previous teaching had focused on 'doing' statistics, including an emphasis on the mathematics underpinning particular methodologies, with little emphasis on its contextualization. However, it was felt that, in line with the recommendations of the GMC, the undergraduate medical students were more likely to become 'consumers' or users of statistics. Thus, they would need to be able to interpret research findings and explain basic concepts such as risk to patients. As a consequence it was decided to concentrate the teaching on ensuring that students had an understanding of how to interpret the basic statistical concepts that they would encounter in the literature and in patient information leaflets. The amount of formulae that they encountered was minimized and little time was spent covering the mechanics of individual statistical tests.

The project aims and objectives were set in terms of both educational aspirations and the production of new materials. The goals of the project were to:

• ensure that students were equipped with skills in interpreting data that would enhance their future performance as doctors

• improve student awareness of the growing importance of medical statistics in medical practice

• improve students' ability to use data to inform their medical practice, thus arriving at greater understanding of the underlying statistical principles

• increase the amount of learner-centeredness in appreciating the role of statistics

• develop materials that allowed students flexible access, and more opportunities to interact with materials at their own pace

• provide materials giving support and feedback to students at various levels, and allowing students to interact with materials until a deeper level of understanding was achieved. Extra or 'extension' work would be a feature for those who needed it.

Although there had been previous revamps to the module, with an increasingly diverse and large student body statistics was still failing to touch many students. Ways of addressing this were sought, so that the revamped approach would provide real alternatives for learning, both for students who had previously studied maths/statistics at High school diploma level and for those who had not. A number of factors were identified that the literature suggested could be helpful to learners, such as the use of medical examples, the use of a variety of visual forms and the provision of an online learning resource for students to access materials repeatedly [8,11-14]. Importantly, materials were created that presented the same information in a number of ways, maximizing the opportunities for students with different learning styles and approaches.

The one hour lecture session was kept as the main form of input, and the approach of using a statistician to present the statistics and a clinician to contextualize them, was introduced as this appeared to hold promise particularly with respect to the contextualization of learning. As previous student feedback from other part of the degree course suggested that in addition to large lectures, the students liked small group tutorials, each lecture was followed up by small group tutorial sessions with 8 students to a tutor. As there were 260 students per year this required approximately 16 faculty giving the same tutorial twice. Not all tutors were statisticians, but all were either statistics PhD students or quantitative researchers with several years experience. An information session was held prior to the commencement of teaching to discuss the main points to be covered in the tutorials and answer any queries that the facilitators might have. During these tutorials the concepts introduced in the lectures were reinforced by means of problem solving exercises based on the medical literature. After significant discussion within the project team, some new additions were made to the teaching:

• Each of the five lectures would open with a short dramatised video-clip designed to contextualise statistical methods being investigated in that lecture. Each clip depicted a scenario based in a family practitioner's office, and set up a realistic problem that required the use of applied statistical methods in order for it to be resolved. These scenarios featured a trainee family practitioner, chosen to be someone the students might be able to relate to tackling problems that they were likely to encounter in their everyday practice. For example, in one of the scenarios the trainee was initially consulted by an elderly patient with asthma who was on a range of medications to treat it. Following a discussion with one of the other doctors in the practice she decided to look at a recent research paper investigating the impact of stepping down therapy. A second video clip at the end of each lecture showed how the fictitious characters resolved the issues and how statistics played a vital role in diagnosis and treatment. In the example above, having looked at the evidence as presented in the research paper the trainee decided that it would be fine to reduce therapy for the patient concerned. Each 'learning episode' was carefully considered in order that the topic of the week was addressed, by applying the relevant statistical approach to the process of making medical judgements. The video scenarios were informed through a number of consultations both with clinicians working in family practice and medical statisticians, in order that they accurately reflected both medical and statistical perspectives.

• A number of animations were created to illustrate certain concepts that benefited from the dynamic explanations made possible by this medium, and that were known to be found difficult by students. These also had the additional benefit of providing breaks in the time spent lecturing and a change of pace, so as to improve student concentration and attention.

### Statistical Methods

In order to evaluate the new teaching approach and compare it with the old one an evaluation questionnaire was distributed to students at the end of the final teaching session, for two cohorts: the last cohort to be taught using the old approach and the first cohort to be taught using the new approach. Ethics approval for this was sought and but as it was regarded as a teaching evaluation in line with standard departmental teaching policy full ethics approval was not deemed necessary. The final teaching session occurred at the same point in the academic calendar for both cohorts. Every student who attended the final statistics session for both cohorts was asked to fill in the evaluation questionnaire and hand it in as they left the lecture theatre. The questionnaires were all anonymous and completion was not compulsory. As completion was anonymous, no attempt was made to link individual student responses to grades. In addition to collecting basic data on the students including their age, sex and previous level of statistics learning, they were asked to define two of the most fundamental concepts of statistics, a p-value and confidence interval, and answer some questions about their beliefs and attitudes to the subject. These beliefs were measured on a 5-point likert-type scale, from strongly disagree to strongly agree. For each student their ability to define a P-value and a confidence interval was graded from zero (no idea) to 5 (text-book perfect answer) by two of the research team, both of whom had extensive knowledge of statistics. This grading process was undertaken with the assessors blinded to which cohort the students belonged to. Where the individual assessors were unsure of which category value (0 to 5) to assign, agreement was reached after moderation between assessors.

The Chi-squared test with continuity correction (χ_cc_^2^) was used for comparing nominal outcomes. For those variables on an ordinal scale (ordered categories) responses between the two cohorts were initially compared using the Mann-Whitney U test. They were further compared using a multiple regression analysis to adjust for age and sex differences between the cohorts. All analyses were conducted using the SPSS^© ^statistical package. The cut-off for statistical significance was p < 0.05. No adjustments were made for multiple significance testing [15].

## Results

Approximately 260 students are admitted annually onto the medical degree course at our institution. There were 168 respondents for the old approach (old) cohort and 158 respondents for the new approach (new) cohort. There were no significant differences in the mean age (diff: 0.4 years, 95% CI: -0.5 to 1.3 years, p = 0.38) or the previous level of statistical knowledge for the two cohorts (Mann-Whitney U p = 0.34). Whilst there was a higher percentage of male responders in the new cohort (38.9%) compared to the old cohort (29.3%), this difference was not statistically significant (χ_cc_^2 ^p = 0.07) (Table 1).

**Table 1:** Baseline Characteristics of respondents

		Old style teaching (n = 168)	New style teaching (n = 157)
Age	mean (sd)	21.1 (3.7)	20.7 (4.3)
	median (min to max)	20 (19 to 39)	19 (17 to 39)
	% missing	5.3	5.1
			
Sex (%)	male	29.3	38.9
	female	67.3	56.7
	missing	3.6	4.5
			
Level of previous statistics knowledge (%)	none	5.4	5.1
	GCSE (age 16 years)	34.5	33.1
	AS level (age 17 years)	17.9	14.0
	A level (age 18 years)*	39.3	40.8
	Degree (non-statistics)	3.0	7.0

* High school diploma level

**Figure 1:**
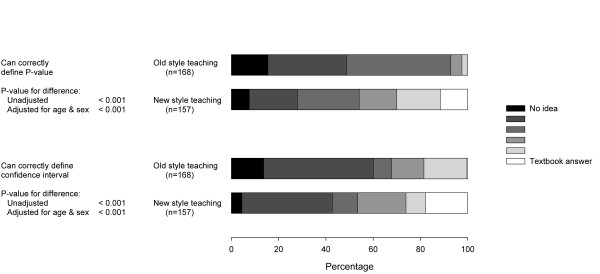
Ability to define key concepts.

Following the teaching there were significant differences between the two cohorts in their ability to define both a P-value and a confidence interval, with students in the new cohort demonstrating an improved ability for both concepts (Figure 1). These differences remained even after adjustment for age and sex. Student ability to define these concepts was graded on a 6 point scale from 0 (no idea or missing) to 5 (textbook definition). After adjustment for age and sex the new cohort scored 1.1 points higher (95% CI: 0.84 to 1.38 points) for the p-value definition and 0.75 points higher (95% CI: 0.42 to 1.09 points) for the confidence interval definition.

**Figure 2:**
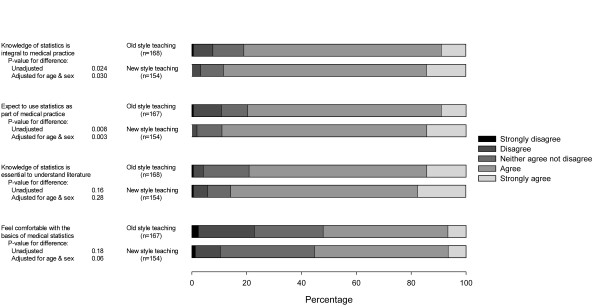
Responses to attitudinal questions.

In addition to their ability to define the two key concepts of p value and confidence interval, students were asked to rate their agreement with four statements about their attitudes towards the relevance of statistics to medical practice. Figure 2 summarises these results. There were significant differences between the two cohorts for their responses to the statements 'A knowledge of statistics is integral to medical practice' (p = 0.02) and 'I expect to use statistics in my medical career' (p = 0.008), with the new cohort being more likely to agree with these statements. These differences persisted after adjustment for age and sex (p = 0.03 and 0.003 respectively). However, there were no significant differences between the cohorts with respect to the two statements 'A knowledge of statistics is essential for me to understand the medical literature' (p = 0.16) and 'I feel comfortable with the basics of medical statistics' (p = 0.18). Adjustment for age and sex did not alter these results (p = 0.28 and 0.06 respectively). Over half of all individuals in both cohorts felt comfortable with the basics of medical statistics with slightly more tending to agree for the new cohort (55.3%) than for the old cohort (52.1%), though as stated above this difference is not statistically significant.

The majority of students in both cohorts felt that the sessions had delivered what they had expected. Though the percentage was higher for the old cohort (74.1% compared to 66.7%) this difference is not statistically significant (Table 2).

**Table 2:** Percentage whose expectations were met?

Old style teaching (n = 162)	New style teaching (n = 138)	Difference (95% CI)	P-value*
74.1	66.7	7.4% (-2.9 to 17.7)	0.20

*Continuity corrected χ^2^

## Discussion

Entry criteria to the MB ChB course have recently been changed to favour entrants from non-traditional backgrounds. Additionally, numbers have risen. The challenge has therefore been to motivate these students to gain a better understanding of statistics in medicine. This is against a background of practice becoming increasingly evidence-based and the requirement on students when they become practitioners to demonstrate that they can reach correct diagnoses using both clinical and statistical data [3,9].

We have described above a project that brought together experts from a range of disciplines to produce a new learning package for medical students. The development of new and remodelled teaching materials and approaches placed greater emphasis on the application of statistics and interpretation of data rather than the mechanics of particular statistical techniques. We evaluated this new approach and demonstrated that we have made a statistically significant difference to both students' understanding of some key concepts, including confidence intervals and P-values, and their ability to view statistics in relation to their medical practice. The new course did place much greater emphasis on obtaining a basic understanding of key concepts and this might in part explain the big difference in the number of students able to define these, although we would argue that this could not account for all the difference between the groups. We did much more than change the emphasis, we completely remodelled the teaching materials and increased the variety of approaches and it is this multi-dimensional approach that we would recommend to others; in order to engage (non-statistics) students in learning about statistics you need to think very carefully about what you want them to learn, make sure you cover the basics well, embed the learning within a relevant context and use a variety of approaches.

Negative attitudes and beliefs towards statistics can impact on how students learn and apply what they have learnt outside the classroom [16]. Introductory statistics courses are important as they may often be the only course that students undertake and they are important in shaping students' beliefs and attitudes [17]. In redesigning the syllabus and the way in which medical statistics is taught we have sought to make the students' learning experience more relevant to their eventual medical practice and ensure that the teaching reflects what is to be learned [10,18].

Although a recent article by Herman et al advocated that medical students be taught techniques in addition to concepts, their conclusion that this resulted in better knowledge retention was not supported by any evidence in their paper [19]. In addition the course they outlined included a large number of contact hours, culminating in a substantial piece of research in the final year. Whilst this might be seen by some as desirable, it is not relevant to the majority of medical degrees where only a limited amount of curriculum time is given over to the teaching of statistics. As has been commented on by several authors, the majority of medical students are likely to become consumers of research and are thus most likely to need to understand basic concepts rather than the details of particular statistical tests and the mathematics that underpin these [9,20-22]. Along with the recommendations of Evans [20] the amount of mathematics taught has been kept to a minimum and the teaching has been rooted in real examples.

Although there have been several calls for changes to medical statistics education so that it is more concept-driven as outlined above, there have been few evaluations of changes to teaching practice for this subject. In addition, as early as 1990, Clayden suggested that collaboration between medical statisticians and non-statistically qualified teachers offered potential benefits for student learning. This current study is the first to do this and to demonstrate the advantages of such an approach. Those that have evaluated new teaching initiatives have tended to concentrate on process and quality issues [21,23], rather than systematically evaluating differences in knowledge between the traditional mathematically orientated teaching compared to concept driven teaching. Only Marantz et al have previously compared outcomes between a more traditional teaching with a new 'case-based' approach that attempts to contextualize learning [11]. They demonstrated that outcomes were comparable between the two and the students were very positive about the new-approach teaching. While the Marantz study differs from this current study in that they used individual cases to illustrate key concepts, its results are in agreement with this current study and reinforces the advantages of contextualized learning.

In addition the Marantz approach did not use the variety of different approaches that the current study has used [11]. We sought to maximize the opportunities for students with different learning styles and approaches using a variety of media and materials, such as lectures, small group tutorials, videos and animations. This is in line with the recommendations of several authors including Nooriafshar & Maraseni who demonstrated that irrespective of background, students preferred visual explanations and recommended that learning be facilitated using a multimedia approach, including animations [13]. The videos that we produced were used to introduce students to the scenarios to be explored during the teaching. While videos have been criticized as a medium for sole instruction, Moore also highlighted its benefits for introducing topics and providing a narrative structure [12]. By increasing the small-group problem-solving component of the teaching we have increased the opportunities for students to explore in greater detail the concepts introduced in the lectures.

We recognise that the work that we have done on this project has relied upon the fact that we have all collaborated well together. This project would not have been as successful had this not been the case and in recommending a collaborative approach to others we would emphasize that this approach will only work if all participants are prepared to work closely together and value the other members of the team. Whilst we have demonstrated improvements in both student attitudes and knowledge, we remain keen to improve knowledge for all and continue to strive to find new ways of improving our teaching, particularly for those who continue to fail to understand. In addition to all that we have done to improve the experience of learning statistics and the understanding that students take away from the sessions, we are currently developing an online resource, as there is a clear need for this [14]. This is designed to serve as an easy reference guide for consolidation of learning and revision. Students will be able to access the content of the lectures, a glossary of terms covered in the lectures, further materials (including links to other web-based statistical resources) and also additional scenarios to contextualise key statistical principles, which include quizzes designed to self-check their knowledge. The resource will also provide more advanced materials for those students interested in delving deeper, beyond the level of the lectures.

In addition, there has been much discussion above of the advantages of contextualising learning, particularly in the learning and teaching of statistics. When we initially began this project, we were keen to use a problem-based learning approach (PBL), but found that given the restrictions of the curriculum, particularly with respect to the time available, it would not be possible at the present. These problems have also been raised by Bland, who investigated the use of PBL to teach statistics in Australian medical schools, [24]. He found that whilst many of the Australian schools used PBL for teaching, there were no instances of a truly integrated approach to the teaching of statistics through PBL. This remains an area for future work that we are now pursuing through the development of PBL materials for our students that fit within the current curriculum.

## Conclusion

We are committed to improving learning, both the student experience of learning and the knowledge that they take away, and it remains our goal that following our teaching the students – who will become doctors – should have more positive attitudes towards statistics, their possible meanings and their impact on the process of consultation. Ultimately we are seeking to make a difference to the skills that students will need when they become medical practitioners dealing with patients. The implications of this learning, therefore, are likely to extend far beyond their time as an undergraduate. This important issue coincides with current wider interest in professional standards. The education and training that medical students receive is fundamental to the formation of their attitudes and behaviours. Doctors increasingly need to use statistics to help them understand medical issues. We are seeking to counter phobias relating to statistics that may exist among students and, by presenting materials in different ways, we have given more students more opportunities to learn important content and principles. It is the pursuit of these professional standards that drives our efforts.

## Competing interests

The authors declare that they have no competing interests.

## Authors' contributions

All authors made substantial contributions to the conception and design of the innovations outlined in the paper, the acquisition of the data and the interpretation of the results of the analysis. All authors helped in revising the draft critically for important intellectual content. All authors approved the final version of the paper prior to submission. In addition JVF was responsible for the analysis of the data and wrote the first draft of the paper.

## Pre-publication history

The pre-publication history for this paper can be accessed here:


